# Comparative Evaluation of Colour Stability and Surface Roughness of Nanohybrid Composite Resins in Mouth Rinse and Colouring Beverages

**DOI:** 10.7759/cureus.35303

**Published:** 2023-02-22

**Authors:** Tribisha Kalita, Chandana Kalita, Lima Das, Rubi Kataki, Lalit C Boruah, Anija R, Anindita Saikia, Putul Mahanta

**Affiliations:** 1 Department of Conservative Dentistry & Endodontics, Siddhartha Dental Clinic & Implant Centre, Guwahati, IND; 2 Department of Conservative Dentistry & Endodontics, Government Dental College, Dibrugarh, IND; 3 Department of Conservative Dentistry & Endodontics, Regional Dental College, Guwahati, IND; 4 Department of Conservative Dentistry & Endodontics, Karewell Dentaire, Pondicherry, IND; 5 Department of Conservative Dentistry & Endodontics, Air Force Station, Barnala, IND; 6 Forensic Medicine and Toxicology, Assam Medical College and Hospital, Dibrugarh, IND

**Keywords:** chlorhexidine, coffee, filtek z 250xt, mouth rinse, surface roughness, colour stability, composite resins

## Abstract

Objectives

An ideal restorative material should be capable of supplanting the biological, functional, and aesthetic qualities of a healthy tooth structure. There has always been a search for optimal and aesthetically pleasing restorative materials. This study aims to evaluate the surface roughness and colour stability of three nanohybrid composite resins post-exposure to mouth rinse and colouring beverages.

Methods

One hundred and twenty specimens of dimension 10 mm x 8 mm x 1 mm were randomly allocated into three equal-sized groups and fabricated using three different nanohybrid composites (Group A: Filtek Z250 XT, Group B: Tetric N-Ceram, and Group C: Solare Sculpt). Sixty samples, comprising 20 from each group, were examined for colour stability and 60 for surface roughness after exposure to chlorhexidine and coffee. Baseline and post-exposure readings of the surface roughness and colour absorbance of the specimens were obtained by atomic force microscopy and spectrophotometer, respectively. A one-way analysis of variance (ANOVA) test followed by a post hoc Tukey's test and an independent t-test were used for data analysis, considering a p-value<0.05 as significant.

Results

Irrespective of the composite, the surface roughness and colour change were substantially higher in the samples exposed to coffee (p-value<0.01). Filtek Z 250XT showed significantly minor changes in colour and surface roughness, followed by Solare Sculpt and Tetric N-Ceram (p-value<0.05).

Conclusion

Coffee caused more surface roughness and colour changes compared to chlorhexidine. Filtek 250 XT showed minor changes in colour and surface roughness on exposure to both solutions.

## Introduction

A quest for an ideal and aesthetically acceptable restorative material is probably as old as dentistry itself, and the development of composite resins has enabled patients' consciousness of self-aesthetics. Nanohybrid composites contain both conventional and nanometric fillers that make the material resistant to changes in colour, aesthetically pleasing, and highly polishable [1,2].

Colour stability is the most desirable property of materials used for aesthetic restoration; therefore, maintaining the colour is of utmost importance. Various internal or external factors may affect the colour stability of dental materials. Intrinsic factors like chemical changes in the material due to oxidation processes and saturation of colourants from external sources such as mouth rinses, coffee, tea, and nicotine may result in discolouration of the material [3]. Moreover, the degree of staining depends on the hydrogen ion concentration of the solution exposed, the type and constituents of the composite, and the finishing and polishing modalities [4].

Colour stability and surface characteristics of composites concerning texture, topography, and roughness are correlated, as rough surfaces lead to greater plaque retention and make the surface more susceptible to discolouration than a relatively smooth surface [5]. Therefore, this study compares the effects of chlorhexidine and coffee on the colour stability and surface roughness of three different nanohybrid composites using recent imaging technologies like UV spectroscopy and atomic force microscopy (AFM).

## Materials and methods

This in vitro study was conducted in the Department of Conservative Dentistry and Endodontics, Regional Dental College, Guwahati, Assam. The resin composite samples were tested in the Department of Central Instruments Facility, Indian Institute of Technology Guwahati, Assam, and the Department of Biochemistry, Gauhati Medical College. The study approval was obtained from the Institutional Ethical Committee of the Regional Dental College, vide Ref No. RDC/29/2011/1622.

The study samples consisted of 120 resin-based composite materials fabricated in the Department of Conservative Dentistry and Endodontics, Regional Dental College, Guwahati. Plastic moulds of size 10 mm x 8 mm x 1 mm were used to fabricate the samples using the nanohybrid composites under study. The samples were randomly allocated into three different groups of size 40 each: Group A: Filtek Z250 XT (3M ESPE, Seefeld, Germany); Group B: Tetric N-Ceram (Ivoclar Vivadent, Liechtenstein); and Group C: Solare Sculpt (GC Dental Products Corp., Japan), based on the nanohybrid composites used.

Evaluation of Colour Stability and Surface Roughness

For colour stability evaluation, 60 prepared samples (20 samples from each group's A, B, and C) were immersed in distilled water in separate plastic beakers as per the group, respectively, at room temperature for 24 hours. After 24 hours, the baseline colour values of samples were recorded with a UV spectrophotometer (V-770 UV-Visible/NIR Spectrophotometer, Easton, Maryland, USA).

After baseline colour measurement, the experimentation was continued by immersing ten samples of each group in 20 ml of 0.2% chlorhexidine and ten samples of each group in 20 ml of coffee solutions (prepared by adding 4 g of coffee powder to 300 ml of boiling water and then boiling the solution for five minutes) for five seconds, and then in artificial saliva for five seconds for a total of five minutes in the morning and five minutes in the evening at a fixed time every day and storing the remaining time in artificial saliva for the remaining time to simulate a person's mouth rinse usage on average. The cycle was done for 28 days. Samples for both solutions were washed and brushed daily using a powered toothbrush for two minutes to remove any debris. The beverages were replenished and prepared daily using the same amount for standardisation [6]. After 30 days, the samples were washed with distilled water and blotted dry before being subjected to UV spectrophotometric analysis for the detection of colour change, if it occurred.

For evaluation of surface roughness, the remaining 60 samples, comprising 20 from each group, were treated following the same procedure. Then each treated sample was studied under an atomic force microscope (Oxford Instruments; model: Cypher), and the post-immersion images were documented for individual samples [6]. The protocol continued in the study was to immerse the samples in the solutions for five minutes each in the morning and evening for 28 days, equivalent to two minutes of use daily for two years [7].

Statistical Analysis

The comparison of baseline and post-exposure data were made using the one-way analysis of variance (ANOVA) and Tukey's post-hoc test, considering a p-value <0.01 as significant. The IBM Statistical Package for Social Science (SPSS) version 20 was used for data analysis.

## Results

Irrespective of the composite considered, the mean absorbance was significantly higher post-exposure to coffee than chlorhexidine. Tetric had the highest mean colour absorbance (1.575 ± 0.254) (absorbance unit) post-exposure to coffee. On the other hand, Filtek had the lowest mean colour absorbance (0.302 ± 0.039) (absorbance unit) post-exposure to chlorhexidine (Table 1).

**Table 1:** Comparison of the mean colour absorbance of the composites #p-value <0.05, the test result is statistically significant

Composite	Solution	N	Mean	Standard deviation	t-test value	p-value
Filtek	Chlorhexidine	10	0.302	0.039	10.464	<0.001^#^
	Coffee	10	1.209	0.153		
Solare	Chlorhexidine	10	0.345	0.077	17.092	< 0.001^#^
	Coffee	10	1.313	0.032		
Tetric	Chlorhexidine	10	0.360	0.032	11.639	<0.001^#^
	Coffee	10	1.575	0.254		

Concerning chlorhexidine, the colour absorbance was found to be lowest in Filtek compared to Solare and Tetric. However, the difference was not statistically significant (p-value>0.05). On the other hand, when exposed to coffee, absorbance was found to be lowest in Filtek compared to Solare and Tetric. No significant difference in mean colour absorbance between Filtek and Solare groups was noted (p-value>0.05). But the mean colour absorbance of Tetric when exposed to coffee was significantly higher than that of both Filtek and Solare (Table 2).

**Table 2:** Post hoc comparison of the mean colour absorbance of the three composites #p-value <0.05, the test result is statistically significant

Solution	Composite	N	Mean absorbance	Standard deviation	ANOVA test value and p-value	Tukey's post hoc analysis		
								Chlorhexidine	Filtek	10	0.302	0.039	3.186, p = 0.057	Filtek vs. Solare	p = 0.188	
Solare	10	0.345	0.077	Filtek vs.Tetric	p = 0.055								
Tetric	10	0.360	0.032	Solare vs.Tetric	p = 0.806								
Coffee	Filtek	10	1.209	0.153	11.94, p<0.001^#^	Filtek vs.Solare	p = 0.372	
	Solare	10	1.313	0.032		Filtek vs.Tetric	p <0.001^#^	
	Tetric	10	1.575	0.254		Solare vs.Tetric	p = 0.006^#^	

As seen in Table 3, surface roughness was significantly higher when exposed to coffee than to chlorhexidine in all three composites (p-value<0.05). Filtek showed the least surface roughness compared to the other two composites upon exposure to chlorhexidine and coffee. In contrast, Tetric was observed to have the highest mean surface roughness when exposed to both solutions.

**Table 3:** Comparison of the mean surface roughness of the composites #p-value <0.05, the test result is statistically significant

Composite	Solution	N	Mean roughness	Standard deviation	t-test value	p-value
Filtek	Chlorhexidine	10	3.49	1.14	10.612	<0.001^#^
	Coffee	10	7.85	0.62		
Solare	Chlorhexidine	10	4.43	1.71	29.487	<0.001^#^
	Coffee	10	25.40	1.46		
Tetric	Chlorhexidine	10	6.61	2.55	15.853	<0.001^#^
	Coffee	10	67.12	11.80		

The lowest surface roughness was noted in the Filtek group compared to Solare and Tetric while exposed to coffee and chlorhexidine. The differences were observed to be statistically significant (Table 4).

**Table 4:** Post hoc analysis of the mean surface roughness of the composites #p-value <0.05, the test result is statistically significant

Solution	Composite	N	Mean roughness	Standard deviation	ANOVA test value (p-value)	Tukey's post hoc analysis		
								Chlorhexidine	Filtek	10	3.49	1.14	7.152 (p=0.003^#^)	Filtek Vs Solare	p = 0.515	
Solare	10	4.43	1.71	Filtek Vs Tetric	p = 0.003^#^								
Tetric	10	6.61	2.55	Solare Vs Tetric	p = 0.041^#^								
Coffee	Filtek	10	7.85	0.62	196.30 (p<0.001^#^)	Filtek Vs Solare	p < 0.001^#^	
	Solare	10	25.40	1.46		Filtek Vs Tetric	p < 0.001^#^	
	Tetric	10	67.12	11.8		Solare Vs Tetric	p < 0.001^#^	

## Discussion

The colour stability of composites is essential for both patients and dental practitioners, as the effectiveness of composite restorations depends on it. Staining and discolouration are the main causes of replacing tooth-coloured restorative materials. Discolouration occurs due to various extrinsic or intrinsic variables. Intrinsic discolouration can happen due to material ageing, including changes to the resin matrix, the matrix interface, and fillers. Extrinsic discolouration can happen when colourants from exogenous sources such as coffee, tea, tobacco, drinks, and mouth rinses are absorbed or adsorbed.

Composite resin is composed of matrix, filler particles, and a silane coupling agent. A recent advancement, including the addition of nano-sized fillers to the resin matrix, provides composite resin with the handling properties and polishability of a micro-filled composite in addition to the strength and wear resistance of a traditional hybrid composite resin. Parameters like colour stability and surface roughness of composites depend on the type of organic matrix, the size and distribution of filler particles, and the exposure of the material to low-PH foods, drinks, and mouthwash solutions. The present study evaluated the effects of mouth rinses containing chlorhexidine and coffee on three nanohybrid composites' colour stability and surface roughness.

The nanohybrid composites used in the study were Tetric N-Ceram, Solare Sculpt, and Filtek Z 250 XT. All three composites have matrices with different sizes and loadings of filler particles. All three materials can be used for class I, II, III, IV, and V cavities as direct restoratives for wedge-shaped defects and root surface cavities, core build-ups, direct restoratives for veneers, and diastema closure.

Chlorhexidine gluconate is a cationic antiseptic agent, and mouthwash is generally prescribed for its bactericidal properties. It is frequently used in colour stability studies involving mouthwashes. Numerous studies have reported that exposure to chlorhexidine increased the surface roughness of the curative materials more than storage in artificial saliva. This could be due to the difference in pH values between chlorhexidine and artificial saliva. Similarly, coffee is a stronger chromatogen. Coffee also contains a large amount of staining agents like gallic acid, which may be associated with increased surface roughness due to its acidic pH of approximately five.

**Figure 1:**
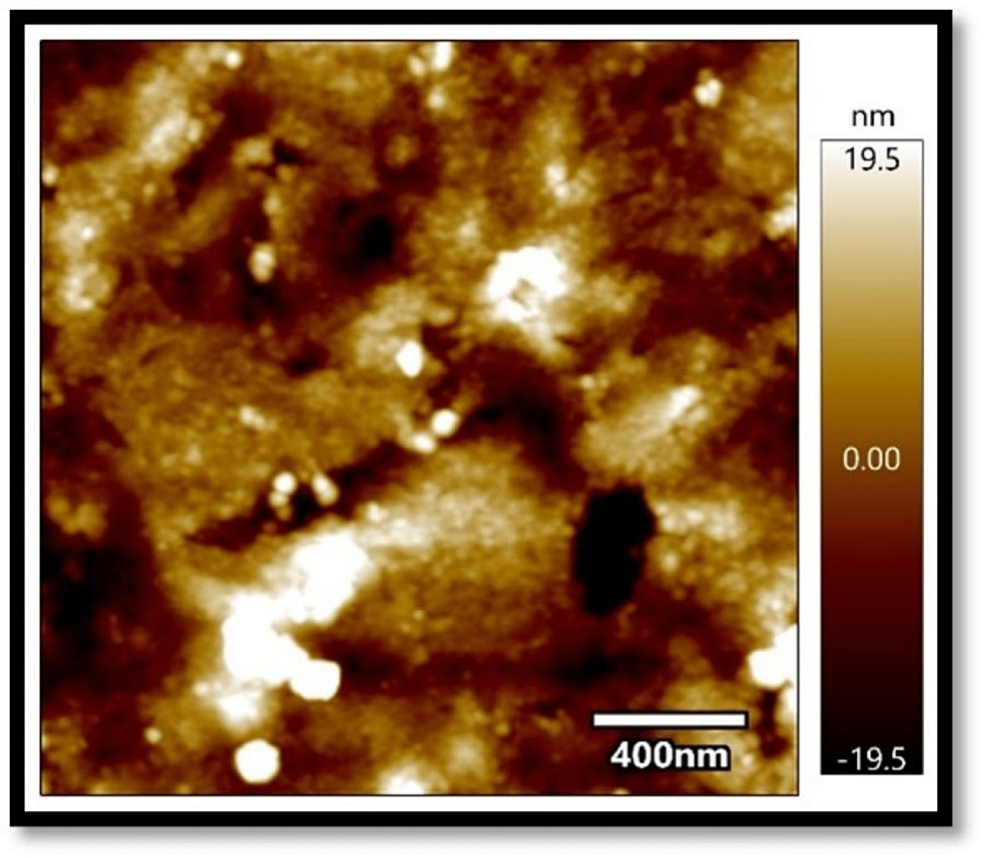
Atomic force microscopy images of Filtek Z250 XT on exposure to coffee

**Figure 2:**
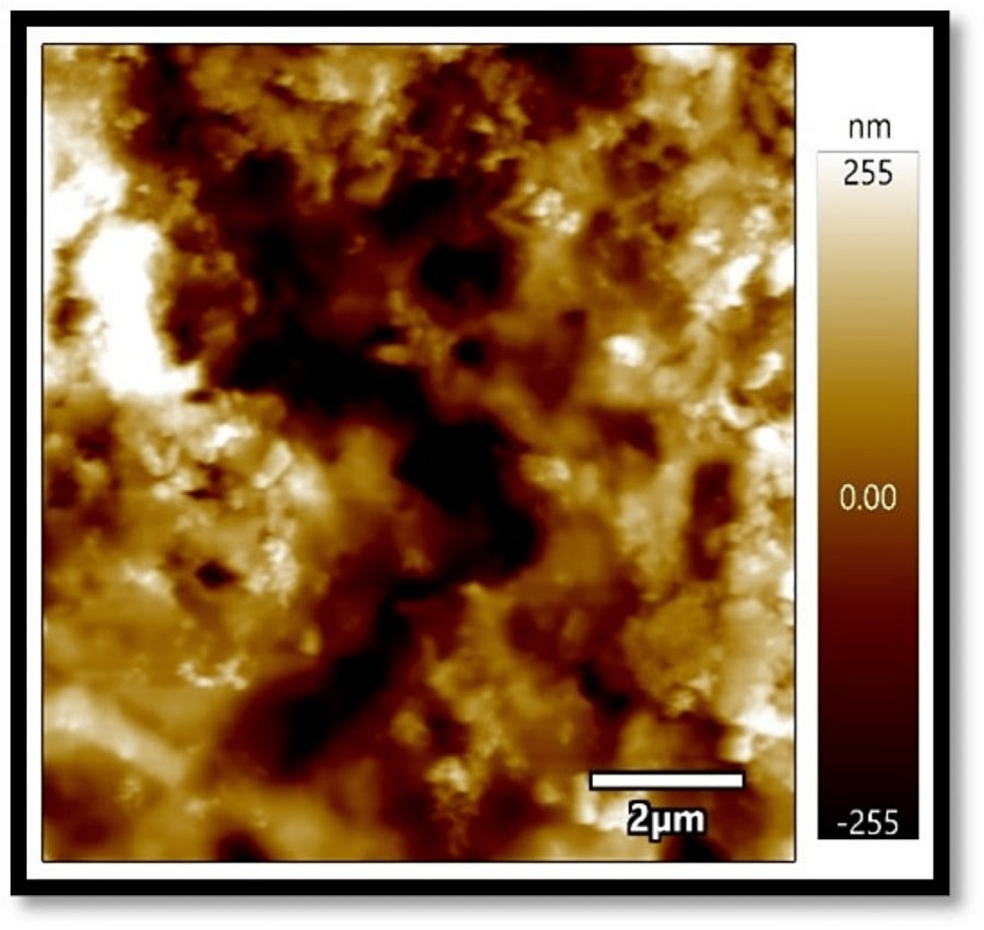
Atomic force microscopy images of Solare Sculpt when exposed to coffee

**Figure 3:**
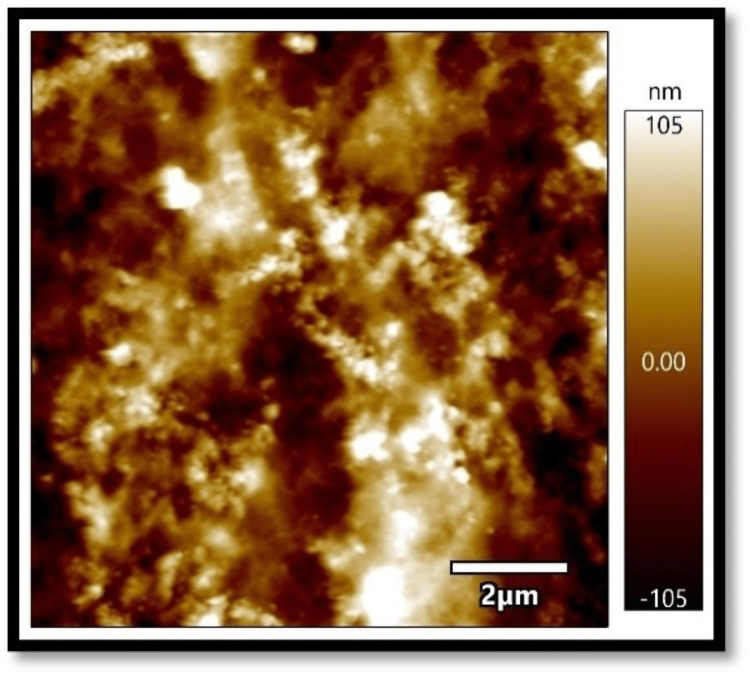
Atomic force microscopy images of Tetric N-Ceram on exposure to coffee

**Figure 4:**
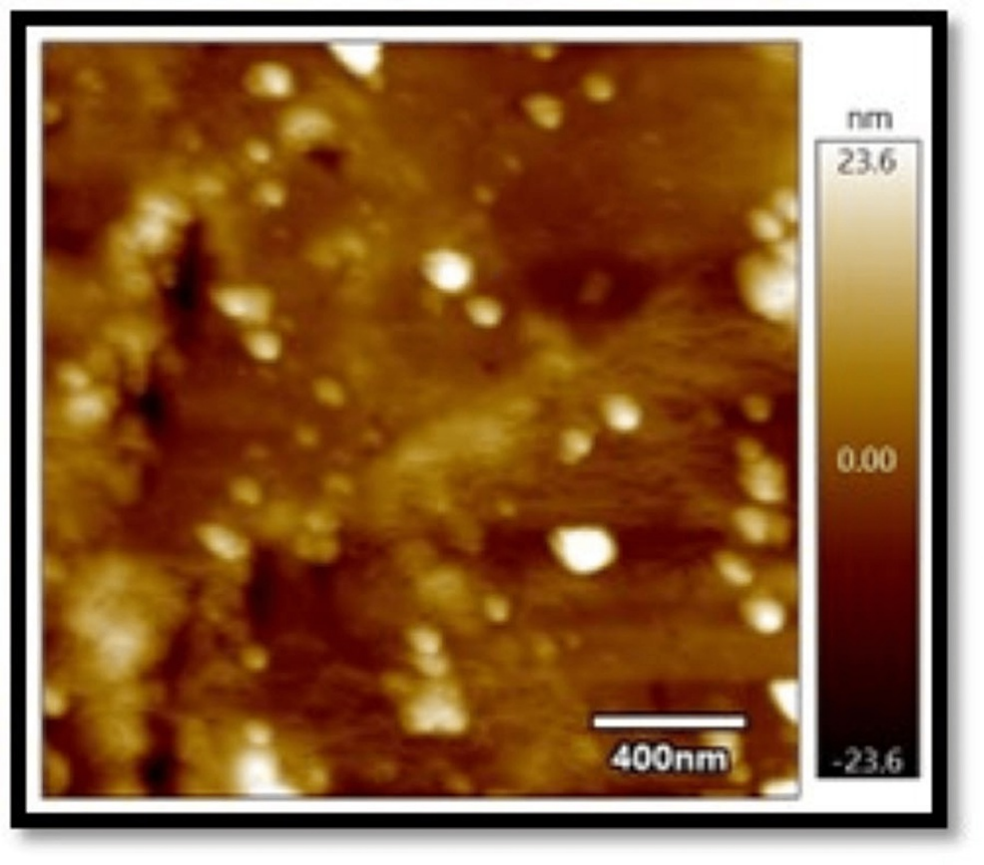
Atomic force microscopy images of Filtek Z250 XT on exposure to chlorhexidine

**Figure 5:**
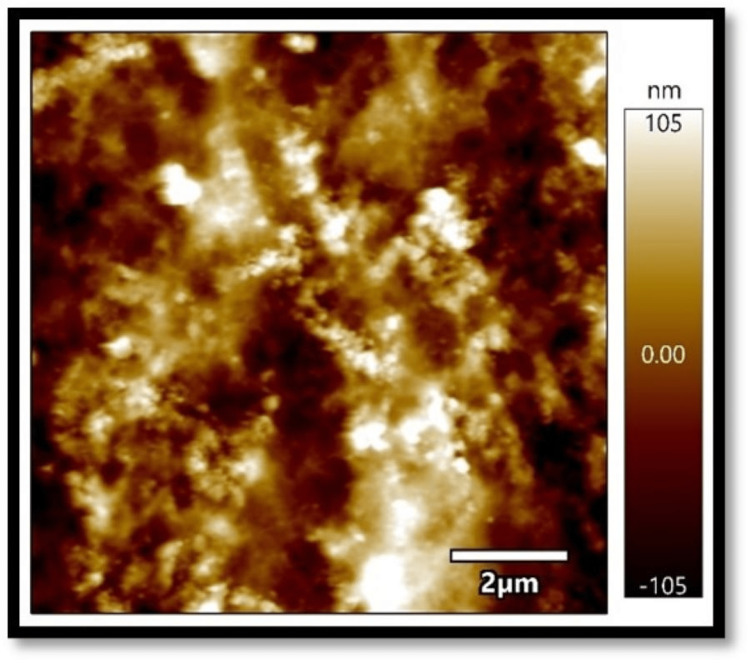
Atomic force microscopy images of Tetric N-Ceram on exposure to chlorhexidine

**Figure 6:**
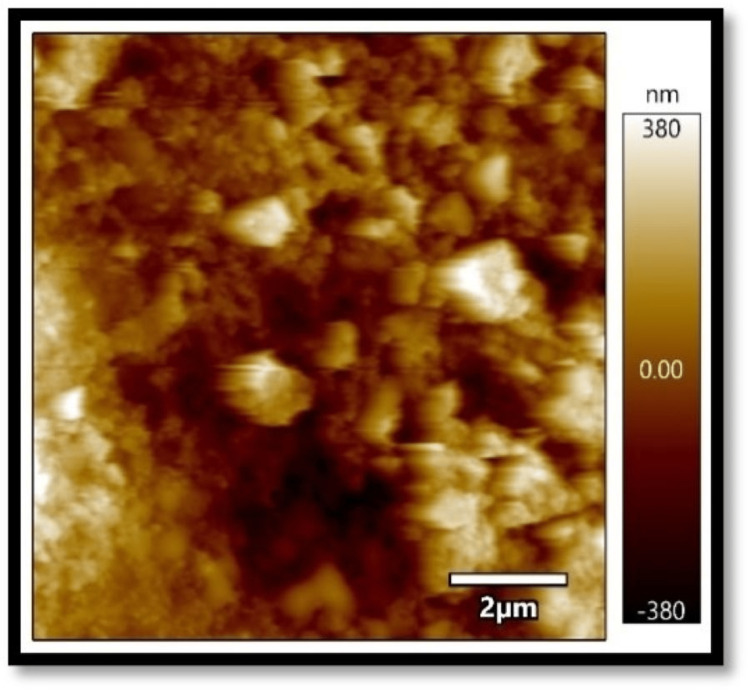
Atomic force microscopy images of Solare Sculpt when exposed to chlorhexidine

In all three composites, the mean absorbance in the coffee group, as shown in Figures 1-3, was found to be higher than that in the chlorhexidine group, as shown in Figures 4-6.

The differences in mean absorbance values between chlorhexidine and coffee were statistically significant, irrespective of the composite used (p<0.001). Also, the mean surface roughness in the coffee group was found to be higher than that in the chlorhexidine group. The differences in mean surface roughness values between chlorhexidine and coffee were highly significant (p-value<0.001) irrespective of the composite used.

It was found in the present study that, irrespective of the composites considered, the mean absorbance and surface roughness caused by coffee (dark coffee) were higher compared to chlorhexidine. The pH of chlorhexidine and coffee are 5.5-7 and 4.5-5.10, respectively. Acidic pH causes hydrolysis of ester radicals present in the dimethacrylate monomers, such as bisphenol A glycidyl methacrylate (Bis-GMA), ethoxylated bisphenol A dimethacrylate (Bis-EMA), urethane dimethacrylate (UDMA), and triethylene glycol dimethacrylate (TEGDMA), resulting in probable degradation of the composites. Lower pH might damage resin composites' surface integrity, leading to more surface roughness and absorbance and less colour stability [8-13].

Also, the present results showed an association between surface roughness and colour change when exposed to the solutions. Filtek showed the least surface roughness and colour changes among the three composite resins under study when exposed to the experimental procedure. Several studies reported a positive correlation between surface roughness and the colour change of the composites [14,15]. On the contrary, Ghinea R et al., in their study, found no linear correlation between surface roughness, chrome, and hue [16].

All three composite resins under study were nanohybrid composites. Still, the colour stability and surface roughness depicted by each of the materials were different, which may be due to the difference in the structure of the resin matrix and the size and constituents of the fillers in composites.

In Filtek Z 250 XT, the monomer matrix is composed of tetraethyleneglycol dimethacrylate (TEGDMA), polyethylene glycol dimethacrylate (PEGDMA), aromatic urethane dimethacrylate (AUDMA), urethane dimethacrylate (UDMA), and 1,12-dodecane dimethacrylate (DDDMA). The fillers include non-agglomerated or non-aggregated 20 nm silica, non-agglomerated or non-aggregated 4-11 nm zirconia, aggregated zirconia/silica cluster filler (composed of 20 nm silica and 4-11 nm zirconia particles), and YbF3 filler made up of agglomerated 100 nm particles. The amount of inorganic filler is around 82% by weight (68% by volume).

While dimethacrylates account for 19%-21% of the matrix in Tetric N-Ceram, almost 53%-55% of the total volume or 75%-77% of the weight comprises inorganic fillers. Prepolymer, ytterbium fluoride (YbF3), mixed oxide, and barium glass make up the fillers. Catalysts, stabilisers, pigments, and other ingredients comprise the remaining 1.0% of the weight. The inorganic fillers have particles between 0.04 and 3 µm in size, and the particles are 600 nm in size on average.

The third composite resin under study is Solare Sculpt, which contains homogeneous, pre-polymerized nano-fillers with high density and uniform dispersion silane treatment technology and 300-nm strontium glass fillers homogeneously dispersed for high flexural strength and wear resistance.

The difference in colour stability and surface roughness among the nanohybrid composites under study can be explained by the fact that smaller fillers and higher filler content reduce the organic matrix, resulting in better distribution of fillers within the matrix, which improves the surface smoothness and colour constancy of the composite resin. Thus, the staining vulnerability of composites is not related to external causes such as surface roughness alone but to inherent influences such as filler and matrix composition [17-20].

Resin material incorporating urethane dimethacrylate is more stain-resistant than dimethacrylate [21,22]. The absorption of fluids may also cause discolouration of the resin composites by the silane, which is again connected to the resin ingredient of composites and the quality of interaction between the resin and the filler [23]. Due to water absorption by the polymer, coupling agents cause hydrolysis and the loss of chemical bonds between the resin medium and the filler units [18]. This leads to surface roughness as filler particles dislodge from the material's outer surface. If there is excessive water absorption, the resin ingredients get plasticized through hydrolysation and microcrack formation. Consequently, the interface between the matrix and fillers allows discolouration [24,25].

## Conclusions

The study's findings suggest that irrespective of the composite considered, the surface roughness and the colour change were significantly higher in the samples exposed to coffee. Filtek Z 250XT showed significantly least changes in colour and surface roughness, followed by Solare Sculpt and Tetric N-Ceram. Within the limitations of the study, it can be noted that the solution's acidity affects the colour stability and surface roughness of the material being exposed. Also, colour stability and surface roughness depend on the resin matrix's configuration, filler content, size, and distribution. Furthermore, greater surface roughness results in more colour change.
